# Collaboration for Impact: Co-creating a Workforce Development Toolkit Using an Arts-based Approach

**DOI:** 10.5334/ijic.5377

**Published:** 2020-06-09

**Authors:** Juliet Rayment, Manbinder Sidhu, Polly Wright, Patrick Brown, Sheila Greenfield, Stephen Jeffreys, Nicola Gale

**Affiliations:** 1 Centre for Maternal and Child Health Research, City, University of London, GB; 2 Health Services Management Centre, University of Birmingham, GB; 3 The Hearth Centre, GB; 4 Sociology, University of Amsterdam, NL; 5 Medical Sociology, Institute of Applied Health Research, University of Birmingham, GB; 6 Suresearch Mental Health Network, GB; 7 Health Sociology and Policy, Health Services Management Centre, University of Birmingham, GB

**Keywords:** Risk, arts-based methods, community health workers, collaboration, workforce

## Abstract

**Introduction:**

The identification, communication and management of health risk is a core task of Community Health Workers who operate at the boundaries of community and primary care, often through not-for-profit community interest companies. However, there are few opportunities or resources for workforce development. Publicly funded researchers have an obligation to be useful to the public and furthermore, university funding is increasingly contingent on demonstrating the social impact of academic research. Collaborative work with participants and other stakeholders can have reciprocal benefits to all but may be daunting to some researchers, unused to such approaches.

**Methods:**

This case study is an account of the co-creation of a (freely accessible) workforce development toolkit, as part of a collaboration between academics, community interest companies, patients and services users and arts practitioners.

**Results:**

Our collaborative group produced three short films, fictionalising encounters between Community Health Workers and their clients. These were used within a series of five discussion-led workshops with facilitator guidance to explore issues generated by the films. Two collaborating community-based, not-for-profit organisations piloted the toolkit before its launch.

**Conclusion:**

We aim to encourage other academics to maximise the impact of their own research through collaborative projects with those outside of academia, including research participants and to consider the potential value of arts-based approaches to explore and facilitate reflection on complex tasks and tensions that make up daily work practices. Whilst publication of findings from such projects may be commonplace, accounts of the process are unusual. This detailed account highlights some of the benefits and challenges involved.

## Introduction

Community health workers have, over the last 25 years, becoming increasingly recognised as an important part of the integrated health/care workforce globally [1,2] by performing outreach and embodying a cultural link between health and care systems and the wider community. Community health workers can be understood as a type of ‘boundary spanner’ [3,4], which operate outside of or in tension with traditional professional hierarchies and systems. They often have little organisational authority, yet are required to bridge knowledge and build relationships across different systems (such as health and social care) [5]. There can be significant costs to the individual brokers who may be overwhelmed and need support [3]. Community Health Workers are certainly often politically marginal within the systems they serve; they may not receive (much) recompense for their work, or be given much opportunity to undertake continuing professional development or receive supervision. The growing use of Community Health Workers in Europe and other high income countries can be understood as an intervention which is part of a broader integrative strategy for the local health economy rather than a simple public health initiative for managing chronic health conditions. However, for such integrated interventions to work, it is essential to create spaces for building human resource.

This case study presents an innovative example, using arts-based approaches, of a way of working collaboratively to offer support for to this workforce that is – crucially – informed by evidence. We present a detailed account of the co-creation of a workforce development toolkit on risk work for Community Health Workers, through the translation of academic theory (generated by empirical research) into a practical intervention. The toolkit was developed by a collaborative group of community health practitioners, academics, service user-researchers and creative professionals and comprised five, in-house workshops with detailed facilitator guidance. The workshops, films and accompanying materials can be downloaded for free from www.birmingham.ac.uk/riskwork.

Community Health Workers are trained to support clients, often from within their own community, to make lifestyle changes to reduce their risk of developing communicable or non-communicable disease. They provide ‘synthetic social support’ to those who may fall through the gaps of traditional health and care services as well as wider social networks, which as well as practical support and advice includes signposting to relevant services and co-ordinating care [6]. As a human resource ‘tool’ of integration, implementing interventions based on epidemiological evidence about ‘at risk’ communities, Community Health Workers are often introduced where there is a gap in the provision of health/care, or where members of a community are perceived to have low health literacy or low resilience and unable to access support effectively. Their other integrative role, therefore, is balancing different models of care and treatment based on the one hand on medico-epidemological risk knowledge that drives national prevention agendas and, on the other, more community-based models of care that emphasize resilience [7]. Geographically, in Low and Middle Income Counties (LMICs) their work is often in rural or urban ‘slum’ areas, where it is challenging to recruit or resource medical and social care staff. In High Income Countries (HICs), their use is often targeted to areas with significant health inequalities, where it is perceived that the communities would benefit from building health resilience [7]. In order to carry out their roles effectively, and as relative newcomers to the health and social care landscape, Community Health Workers must learn to explain and justify their role to colleagues and build connections across organisational boundaries [5] while also seeking to build connections and co-produce care packages with patients/clients [8]. Community Health Workers are usually provided with some basic training on risks to health (from a medical perspective) – and lifestyle strategies to reduce those risks. However, they receive little training or supervision for the wider social support roles or their negotiation of the complex health and care systems that they are engaging with. Our toolkit, designed to supplement (not replace) basic training, is designed to be undertaken alongside practice, and aimed to develop Community Health Workers’ reflective skills through facilitated discussion in response to fictional scenarios.

### Collaboration for Impact

Research designed and developed with impact in mind can bring reciprocal benefits to researchers, the public and the quality of academic knowledge [9]. Many health and care services researchers aim to realise research-related, policy, services and societal impacts [10] such as helping to set research priorities and contribute to changing policy, redesigning services and facilitating public education. Developing such contributions and ‘impact’ may require researchers to change research design [11], in order to involve service users in the research team throughout the study. Methods such as theatre may offer a more accessible medium to communicate results to a wider audience [12,13].

Many agree this type of work is valuable, however it may appear daunting to academic researchers, due in part to a lack of guidance on how such projects can be delivered and how to measure their ‘impact’. Furthermore, interdisciplinary working across fields remains rare. Here we provide a detailed case study of the process of one impact project co-created with community interest companies and involving professional artists, with the aim of helping other researchers involved in this type of work and to share the benefits and challenges involved.

### Rationale for the toolkit: Risk Work and Integrated Care

In the context of a ‘risk society’ [14], health care systems are often dominated by risk logics [15] and, consequently, health/care professionals’ interactions with clients often involve forms of risk assessment, management and/or communication driven by population-based studies of human health [16,17]. This may seem at odds epistemologically and practically with the personalised, adaptive principles of integrated care where patient empowerment, care co-ordination, multi-disciplinary teams and individual care plans are key to success [18]. However, interpreting risk during client casework does not merely involve deciding how and when to intervene, but also how to communicate appropriately, who to involve and moreover, taking account of the impact of what is done and said on the practitioner/client relationship [19]. Central to this ‘risk work’ is the interpretation of an organisational understanding of risk(s) facing (or posed by) a particular group (epidemiological knowledge), within the context of an individual case [19], i.e. What does risk, calculated at a population level, mean for the person sitting opposite you in a consultation? In order to achieve this, health workers would ideally work in ways that align with integrated care principles – placing the patient at the centre and delivering services in a co-ordinated and tailored way. However, within the UK model of health service provision, workers’ ability to do this is likely to be inhibited by a lack of whole system working, decision making and accountability [20] making it all the more important to support workers to manage these tensions.

Carrying out ‘risk work’ involves a complex interplay between the worker’s understanding of risk, their efforts to implement an intervention within (to varying extents) an integrated care setting and their relationship with their clients. These three facets: understanding, intervening and relating, can stand in tension with each other. For example, a worker may be trying to build a positive relationship with a client, whilst also identifying and communicating a risk status that may be heavily stigmatising for this client – for example labelling a person as obese [21] or a risk to others [22]. This may, in turn, have implications for care pathways and care co-ordination that may jeopardize the relationship with the client particularly if it requires referral to other agencies.

**Figure 1:**
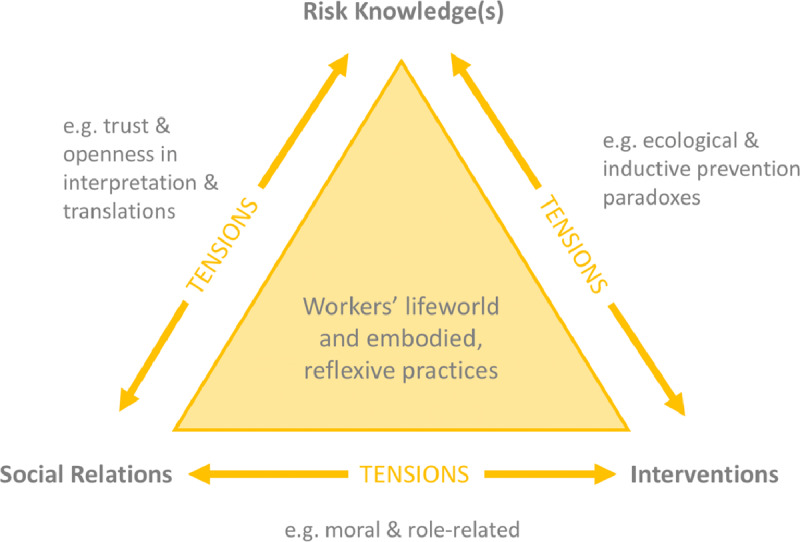
The original model of Risk Work (adapted from [19]).

Figure 1, an output from a research project by the academic authors, captures these combined practices of knowing, intervening and relating, as well as some of the tensions which emerge amid these central features of risk work.

### Community Health Work in the Context of Integrated Care

Horlick-Jones coined the term ‘risk work’ [16], and PB and NG have since built a community of researchers committed to developing theories of risk work [5,6,7,8,19,23,24]. Some of this research has been carried out with Community Health Workers, who can be defined as “individuals with an in-depth understanding of the community culture and language, [who] have received standardised job-related training which is of shorter duration than health professionals, and [whose] primary goal is to provide culturally appropriate health services to the community” [25].

Community Health Work was initially developed to help broaden access to healthcare in rural areas and low-income countries [1], but is now used widely across the world as a cost-effective method of addressing the health needs of high-risk populations [26], including managing non-communicable diseases in high income regions often through care coordination [27].

The role varies depending on local health needs and resources, with some, such as ‘NHS health trainers’, undertaking risk assessments (BMI, blood pressure, Cardiovascular Disease risk scores) with patients, by closely following protocols and algorithms [28,29]. Community Health Workers bring with them nuanced knowledge of cultural beliefs and practices, societal challenges facing the community, and may also share personal experiences of living with an illness and be ethnically or gender matched with patients. They play a crucial role in co-ordinating and integrating care, through supporting clients/patients to engage with various health and social care professionals and supporting their self-management of their condition. Rising super-diversity and the impact on health of social problems such as housing, employment and discrimination, finds increasing number of people who need support from both social work and primary care. Community Health Workers are bridging this gap between services. This can be particularly challenging were there are gaps or failures in integration and relies on the everyday expertise of workers to manage fragmentation, although these kinds of individual efforts are often performed in reactive and idiosyncratic ways, rather than in the context of strategic or planned approaches [30].

In the UK, Community Health Workers are generally employed by Community Interest Companies, who tender for short-term contracts (12–18 months) from local authorities to provide services in a local area. Assessment of commissioned services’ success is often focused on meeting narrow clinical outcome standards (reduction of risk factors associated with particular conditions), rather than the reduction of social risk or wider social or health impact [31,32]. This is at odds with best practice in commissioning integrated care [33,34].

### The challenges of working with risk

Community Health Workers provide health-based work, but within frameworks of risk. While their overall goals is to work within ‘high risk’ communities to reduce the overall burden of disease, their work in practice involves identifying ‘at risk’ individuals and working on a one-to-one basis with them. Qualitative research carried out with Community Health Workers found that they often misunderstood exactly how medical risk was calculated, and were unsure how to explain risks to clients [35]. They were required to build relationships with clients, whilst simultaneously assessing their risk of various health conditions and encouraging them to implement lifestyle changes [17,36]. In addition, Community Health Workers were also acutely aware that when making decisions about health, their clients took into account their social environment and social risks and values as well, such as the importance of hospitality or using certain substances or behaviours to mitigate stressful environments [7].

This research led us to consider how our findings might contribute to supporting Community Health Workers and the community interest companies that employ them. The programme we subsequently developed was designed to help Community Health Workers navigate the complex and challenging tensions within their practice and reflect on the role risk plays in their everyday work, thus supporting third sector organisations and maximising the ‘real world value’ of academic practice for the research participants.

## Method

### Why an arts-based development programme?

Our collaborative approach sought to include stakeholders from the start, particularly Community Health Workers themselves, and their trainers and managers from three local Community Interest companies (these are social enterprises that use their assets and profits for social benefit). The project team also included a survivor-researcher from the Suresearch Mental Health Network with personal experience of using mental health services.

**Figure 2:**
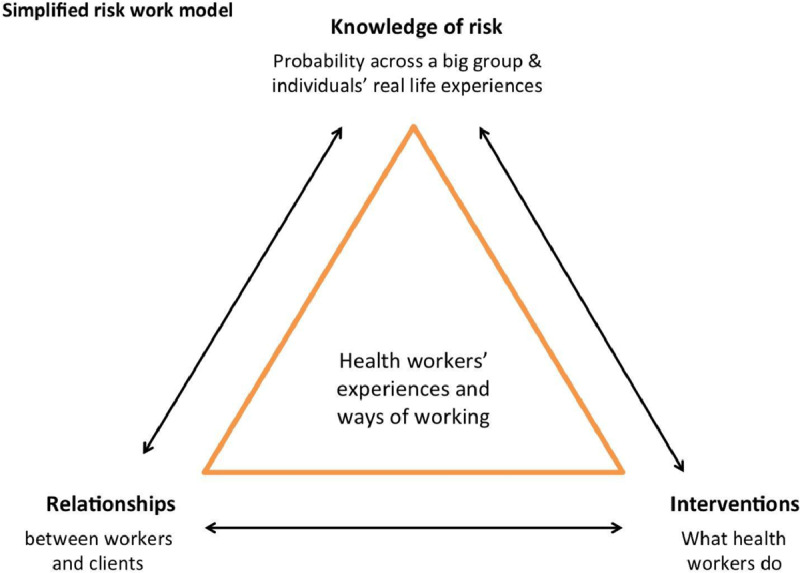
A simplified risk work model.

Two initial exploratory workshops were held with Community Health Workers at two local Community Interest Companies. Using a simplified version of the theoretical risk work model (See Figure 2), and case examples from our research, Community Health Workers shared their own experiences of the different tensions uncovered in our research. This ‘member-checking’ [37] supported our initial research findings and led into a discussion about what an intervention to support these types of organisations, in response to these findings, might look like. Based on their own positive experience of discussing their personal work experiences during the workshops, participants requested a workforce development toolkit to provide a similar experience for colleagues and laid out two key requirements: (i) it should be presented on a digital platform so that it could be used flexibly by organisations (ii) it should avoid a traditional didactic format and aim to stimulate discussion on complex and sometimes difficult topics. Therefore the decision was taken to develop a training programme based around a series of fictionalised encounters between Community Health Workers and clients that raised common issues and tensions that they faced. These scenarios would be filmed using professional actors, therefore reaching a wider audience than live theatrical performances.

### The process: developing a risk work toolkit

Our previous research had generated a theoretical model of risk work (Figure 1) that required some translation for use with a Community Health Worker audience. A Collaborative Group was formed to guide the co-creation of the toolkit throughout the project, helping ensure the end product was user-friendly and relevant. It included a diverse group of academic researchers, Community Health Workers and their managers, service user-researchers, digital training specialists and an arts practitioner. Two external consultants were recruited to take on ‘bridging’ roles between the researcher-academics and the potential users of the toolkit – an arts practitioner (PW), with special expertise in working with literature and theatre within healthcare; and a project ‘Impact Fellow’ (JR), an external academic consultant, with particular skills in facilitation and storytelling.

Creating the toolkit followed six steps:

1. Setting priorities for the toolkit
2. Translating theory and telling the story
3. Developing the format of the workshops
4. Producing and refining the films
5. Piloting and finalising the toolkit
6. Public launch

**Figure 3:**
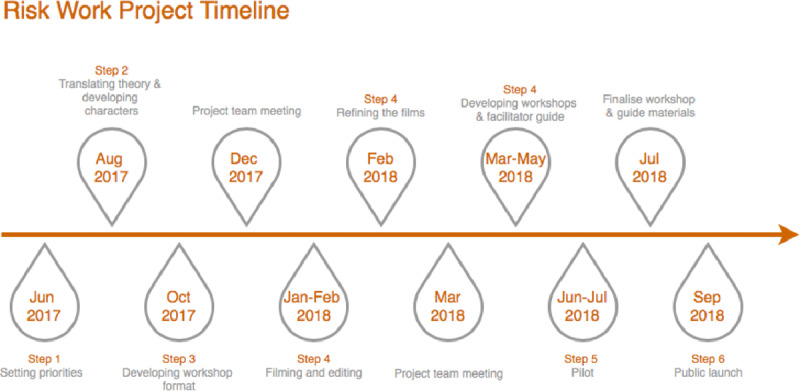
Project timeline.

Figure 3 *shows the timeline for the 15-month-long development of the final training programme*.

#### 1. Setting priorities (Collaborative Group Meeting 1)

At this initial meeting, the group set three core principles for the Workforce Development toolkit:

##### Sustainable

It should be delivered in-house at the Community Interest Companies and not be dependent upon external facilitators or financial support from the University, to ensure continuation beyond the end of the project.

##### Accessible

The toolkit needed to be short and a mix of online and face-to-face learning. A MOOC (Massive Open Online Course) was not deemed appropriate as it was felt that Community Health Workers were unlikely to engage with an entirely online learning environment with formalised and pre-set timescales, but the Group supported an easily accessible visual or interactive format.

##### Supported

Whilst ‘Train the Trainer’ courses would not be sustainable beyond the project end, because of the fixed length of the funding available (from the Economic and Social Research Council), the toolkit should include detailed, free, guidance for facilitators.

#### 2. Translating theory and telling the story (Distillation Day)

The Group had committed to exploring community health work through a series of fictionalised encounters between Community Health Workers and clients. During what we termed the ‘Distillation Day’, the academic team came together with the Impact Fellow (JR) and the playwright (PW) to move the theoretical model (previously generated through academic study of work practices), back to real life everyday Community Health Worker work. We developed characters with which to play out the scenarios and elicited examples of real-life dilemmas faced by Community Health Workers, relating to each side (tensions) of the triangle in the theoretical model (Figure 1). Examples of these included:

##### Knowledge of risk < > Interventions

1. A Health Trainer’s tacit knowledge tells them that signposting doesn’t work because people from that town do not travel to another town access community level support services. How do they adapt guidance to make it realistic?
2. A client’s lived experience doesn’t follow a population trend: for example, their elderly uncle was a regular smoker and lived to be 90.

##### Interventions < > Relationships

1. Community health workers bring their own past experiences and emotional ‘baggage’ to their roles, for example around mental health. This may make it difficult for them to accept it when clients make decisions that clash with their values.
2. Pregnancy outreach workers may be required to advise clients to eat five portions of fruit or vegetables a day, but their clients may be undocumented migrants, living with homelessness or experiencing domestic violence which are more urgent to support them with, making it difficult to focus on delivering health information.

##### Relationships < > Knowledge of risk

1. Health Trainers aim to be non-judgmental but there is stigma around many of the ‘risks’ to vascular health e.g. obesity or smoking. This can impact on relationships as being open about their need to identify risky behaviour or labelling individuals ‘at risk’ is problematic.
2. Community Health Workers may need to break bad news about clients’ risk status. They may not want to create ‘fear’ but need to be honest and transparent about their findings.

##### Developing characters

The group decided the age, gender, ethnicity and role of each character and used a co-writing exercise called Corporate Character [38], to bring the characters to life. Each team member took a turn to ‘hot seat’ a character by answering questions about their character’s imagined everyday life and relationships with their partner, children, best friend, colleague and so on. The characters gradually took shape through fleshing out their relationships with others. The workshops were video recorded for reference. While character development drew on the varied real-life and research experiences of the collaborators, they were entirely fictionalised and not based on any individual research participants. Together we created two pairs of community outreach workers and clients. The first pair explored diet and exercise with a client at high risk of CVD but also experiencing loneliness and bereavement, and the second involved pregnancy outreach work and how cultural assumptions around aspects of identity play into assessments of social risk. A third pair, based on mental health risk, was developed through discussion with the service user-researcher project collaborator who had experience both as a service user and peer supporter in mental health services. Following this, the arts practitioner drafted scripts of three scenarios.

#### 3. Developing the format for the workshops (Collaborative Group Meeting 2)

The full Collaborator Group met to develop the format of the training workshops to accompany the films. These were designed to fit with organisational constraints and need and resulted in a programme of five workshops of one hour each (to fit into scheduled team meeting time). After a performed reading of the draft scripts, the Group suggested amendments and supported the addition of ‘to camera’ monologues for each character to give more insight into their personal thoughts, experiences and motivations.

#### 4. Producing and refining the films (Collaborative Group Meeting 3)

Actors were cast and a professional videographer recruited to film and edit the films. The scenes were recorded out of hours in the university department with locations and props carefully chosen to mimic the fictional settings (a café, community centre meeting room and GP practice consultation room). A fourth film was recorded to introduce key theoretical concepts to the audience through ‘in conversation’ discussions between the project leader and academic colleagues, including project team members. These conversations aimed to use accessible and straightforward language, whilst still challenging course participants to think about risk work in new and critical ways.

The final films were screened to the collaborative group and the feedback was used to develop and refine the workshop materials and facilitator guidance for the pilot. The most vital change made based on the discussions in the collaborative group was further clarity on the scope of the development toolkit, in particular its role as ‘advanced’ training for community health workers with experience rather than initial training for new workers. This arose from a lengthy discussion about some concerns that some of the films showed ‘poor’ practice rather than ‘textbook’ practice. However, others really welcomed the opportunity for open discussions about the challenges of practice and of reflecting on difficulties, so we worked together to steer a path through these frictions that satisfied all collaborators.

#### 5. Piloting the toolkit

The workshops were piloted in two of the three collaborating Community Interest Companies. To replicate ‘real life’ conditions as closely as possible, organisations assigned in-house facilitators and were given the draft Facilitator Guidance and workshop session plans, but no further training from the project team. One facilitator had attended the Collaborator Group meetings; one had not.

To test the practical application of the toolkit, we undertook a small evaluation project which included observation of selected pilot workshops; questionnaires for workshop participants (see Appendix 1) (35 responses) and semi-structured interviews with each facilitator and one manager on their experience of planning and delivering the workshops (Appendix 2). The University Ethics Committee granted an amendment (ERN_17-0466A) to the original ethical approval (ERN_17-0466) for the evaluation observations, questionnaires and interviews.

The sessions were designed to run weekly for five consecutive weeks, but organisational constraints meant some were delayed for longer than a week, and in one case two sessions were held back-to-back. Five out of 10 total sessions were observed by a member of the project team: two held by Organisation A and three by Organisation B. All five sessions were observed (Table 1).

**Table 1:** Observed sessions.

	Organisation	Number of participants	Observed by

Session 1: Challenging our everyday experiences	A	9	JR
Session 2: Up a Gum Tree	B	4	MS
Session 3: Nuts and Seeds	A	9	JR
Session 4: The Devil’s Price	A	9	JR
Session 5: Bringing it all together	B	4	MS

Our key finding from this evaluation was that facilitators often made ‘on the go’ adjustments to the duration or order of workshop activities. Where consistent, these changes were adopted into the workshop timetables. Further changes were made to workshop plans following analysis of questionnaire responses and the facilitators’ reflections during post-delivery interviews – adding in additional clarity where it was requested and altering or offering alternatives for some of the activities.

#### 6. Public launch

We held a public launch event to promote the toolkit, inviting contacts from our academic and practitioner networks, both within Community Health Work and beyond, into nursing and midwifery and other kinds of healthcare work. The launch event was intentionally held outside the University campus in a local arts centre, and included a screening of the three fictional films.

## Feedback and Evaluation: Learning from challenges

A collaborative project like this inevitably faced a number of challenges. However, working to manage or solve these generated lessons that could be applied to other projects of a different topic, aim or scope. Here we describe four main challenges that we identified in the feedback from collaborators and the findings from the formative evaluation.

### Beginning with different priorities → finding overlaps through listening and discussion

We had started with the concept of ‘risk work’ because it was identified as a specific challenge for research participants in the academic team’s previous research – and was selected for funding for impact activity. However, participants at the initial ‘priority setting’ meeting uncovered other equally pressing issues: How do we make sure clients are ready for an intervention? How does short-term commissioning impact on our relationships with clients and the implementation of interventions? How can we better provide supervision for our staff and a space for them to reflect on their work?

The challenge for the collaborative group was to find where their different needs and aims might overlap. As the initial workshops had generated a plan for an arts-based, discussion-led toolkit, we agreed that these sorts of workshops would potentially provide the space and time for staff to experience group support and peer supervision and have an unusual opportunity to reflect and talk about their work thus addressing one of the community health workers’ priority needs in using risk work.

### Need for new approaches to training methods → Arts-based approaches

The Community Interest Companies’ existing training schemes for new Community Health Workers used a more didactic teaching and learning model, focussing on facts and skill development. The programme that we subsequently developed in this project, in providing a reflective and discussion-based method, demanded different facilitation skills and staff engagement. It was at times challenging for members of the collaborative group to differentiate between training that aimed to teach Community Health Workers to improve their skills, compared to reflecting on their practice. For example, whilst the fictionalised films depicted less than ideal practice from Community Health Workers, the aim of the training was not to identify these poor practices, but instead use them to spark discussion on community health work more broadly and participants’ experiences in particular. The challenging discussions in the group meetings where participants fed back about what they perceived as ‘poor’ practice in the films led us to explicitly address these issues when designing the workshop programme and facilitator support documents. The arts-based approaches – and the use of fictionalised encounters between Community Health Workers and clients as a basis for discussion – allowed a sort of emotional ‘distance’ between the characters and the workers enabling them to deal with difficult topics without becoming defensive (cf. Chipatiso 2013).

### Translating a complex theory/model facilitation and storytelling

Figure 1 shows a diagram illustrating the original theoretical model, which required significant time and discussion to explore and work with, not just for Community Health Workers, but also for non-academic research team members, and those academic team members whose expertise is not in risk work. Involving external facilitators and collaborators challenged the research team to find new, more straightforward, ways to explain their ideas and to ‘rewind’ their abstract theory back to the ‘real-life’ from which it had originally been generated to produce fictional accounts that were nonetheless able to capture the essence of the tensions identified by the research.

### Unknown or variable facilitator skills → Detailed facilitator guidance

It was clear from collaborative group discussions that in-house facilitators would need support to deliver workshops that were likely to generate challenging discussions and emotions around work, and work relationships, in an environment that prioritises fact-based teaching. We had no way to know the skills of future facilitators and could assume they would have different levels of skills in managing such discussions. It was also clear that there needed to be no additional costs involved beyond the end of the project. Clear guidance for facilitators was included in the toolkit to help mitigate against these uncertainties and provide consistent information for facilitators. Data collected during the formative evaluation – from observations of sessions and feedback from the facilitators – allowed the team to identify where additional clarity or reframing of facilitator guidance was required.

## Conclusion

This project aimed to apply the findings of academic research to co-create a workforce development toolkit to address particular practice challenges identified by the research. Collaboration brings many different experiences and perspectives to a project, helping to ensure that any toolkit developed is appropriately tailored to its audience. Members of the team were able to draw on a range of personal experiences and identities as they participated in developing the training materials and constructing the characters in the co-writing exercise. Collaboration with Community Health Workers as the end users of the toolkit ensured that it responded to their own organisational priorities; and was in a useable format given the restrictions of their service.

‘Risk’ is a complex topic for any training programme: encompassing issues around interpersonal relationships, as well as probabilistic knowledge, professional judgement, clinical experience and the impact of society and culture all within the context of ‘work’ that may be emotionally challenging or involve varied organisational relationships or insecure employment contracts. As discussed above, in the context of integrated care – and particularly at the interface of medical and social (risk or resilience) models of care – these challenges can be particularly acute because of the epistemological clashes between different forms of knowledge. For organisations accustomed to delivering technical skills-based training, opening up and facilitating discussions on these topics may present new challenges.

In the context of our collaborative approach, we found that using an arts-based approach provided structure for these types of conversations through presentation of the experiences of the ‘other’ in the relationship in ways that are not oversimplified or stereotypical. They functioned in a similar way to vignettes used in qualitative research:

Creating distance between the context of the vignette and the participant, by not asking people directly about their own experiences, rather by asking how third parties might feel, act, or be advised to proceed in a given situation [39].

Structured guidance for facilitators went some way to mitigate against the challenges of providing distance learning programmes through unknown facilitators, by providing consistent information on the aims and structure of each session and on the course as a whole.

The learning about collaboration for impact from this project are, we would argue, likely transferable to other boundary spanning or care coordination roles in the integrated care field – in particular where different forms of knowledge and/or practice norms are in tension with one another within services that remain dominated by biomedical or risk-based logics. The arts-based approach allows for the surfacing of fundamental tensions in ways that are experiences as safer and more supportive – rather than confrontational – by participants.

Developing such a programme through collaboration takes effort, mutual understanding, communication and time. Our community interest company partners were simultaneously managing limited funding and the uncertainty generated by tendering for new contracts. It was essential in this context that our development tool was free to access and could be delivered without the need to buy in facilitation skills. Creating this kind of arts-based intervention as a collaborative endeavour can be a risk in itself, given the challenges of finding shared ways of working, but one that can pay off by ensuring that the participants who dedicate time and energy to the production of academic research can experience its tangible benefits.

## Additional Files

The additional files for this article can be found as follows:

10.5334/ijic.5377.s1 Appendix 1. Participant evaluation questionnaire.

10.5334/ijic.5377.s2 Appendix 2. Facilitator interview topic guide.
