# Supplementary material for: Collaboration for Impact: Co-creating a Workforce Development Toolkit Using an Arts-based Approach
Source: Int J Integr Care. 2020 Jun 9;20(2):11. doi: 10.5334/ijic.5377 (PMC7292144; doi:10.5334/ijic.5377)
Supplement: Appendix 1. — Participant evaluation questionnaire. [file ijic-20-2-5377-s1.pdf]

Appendix 1

Participant evaluation questionnaire

27 April 2018
Version 2

Workshop Participant Feedback Questionnaire for observed sessions

This questionnaire should take no more than 5 minutes to complete. We really value your feedback.

Please tick this box to indicate that you are aware that this questionnaire forms part of the evaluation of this pilot training programme and that you consent to participate □

Please do not write your name anywhere on this sheet as responses are anonymous.

1. What three things did you take away from today's session?

1.
2.
3.

2. How relevant do you think this session was to your work?

Very	Somewhat	Not very	Not at all	I don't know

3. How useful do you think this session will be in your work?

Very	Somewhat	Not very	Not at all	I don't know

5. For which area of your work do you think this is most relevant or useful? Why?

4. What was the highlight of the session?

5. Was there anything you think should have been done differently?

6. Is there anything else you would like to add?

Thank you for participating.
