# Supplementary material for: Collaboration for Impact: Co-creating a Workforce Development Toolkit Using an Arts-based Approach
Source: Int J Integr Care. 2020 Jun 9;20(2):11. doi: 10.5334/ijic.5377 (PMC7292144; doi:10.5334/ijic.5377)
Supplement: Appendix 2. — Facilitator interview topic guide. [file ijic-20-2-5377-s2.pdf]

Appendix 2: Facilitator interview topic guide

23 February 2018
Version 1

Semi-structured interviews with facilitators at the end of the course

Topic Guide

Topics to be covered

1. Expectations of the course
2. Experiences of facilitating including highlights and challenges
3. Practical considerations including organisational facilitators and barriers
4. Recommended changes

Question guide

Can you tell me what it has been like for you to facilitate this course?

What was your expectation of the course?

How much time has it taken you to prepare for the course?

What was the attempting to achieve? How far do you think it achieved that?

What was the highlight for you? Plus prompts around why it worked so well

What did you find challenging? Plus prompts around what could be done differently

How far have the supporting train the trainer materials been useful? How could they be improved?

How far do you feel the organisation has supported this course? Why?

What organisational support has helped? Have there been any barriers within the organisations?

Do you have any suggestions for changes, improvements or adaptations to the course for the future?

Is there anything else you think I should know?
